# Vaccines against human HER2 prevent mammary carcinoma in mice transgenic for human HER2

**DOI:** 10.1186/bcr3602

**Published:** 2014-01-23

**Authors:** Carla De Giovanni, Giordano Nicoletti, Elena Quaglino, Lorena Landuzzi, Arianna Palladini, Marianna Lucia Ianzano, Massimiliano Dall’Ora, Valentina Grosso, Dario Ranieri, Roberta Laranga, Stefania Croci, Augusto Amici, Manuel L Penichet, Manuela Iezzi, Federica Cavallo, Patrizia Nanni, Pier-Luigi Lollini

**Affiliations:** 1 Laboratory of Immunology and Biology of Metastasis, Department of Experimental, Diagnostic and Specialty Medicine, Alma Mater Studiorum University of Bologna, Viale Filopanti 22, I-40126 Bologna, Italy; 2 Interdepartmental Centre for Cancer Research “Giorgio Prodi”, Alma Mater Studiorum University of Bologna, Via Massarenti 9, I-40138 Bologna, Italy; 3 Laboratory of Experimental Oncology, Rizzoli Orthopedic Institute, Via di Barbiano 1/10, I-40136 Bologna, Italy; 4 Department of Molecular Biotechnology and Health Sciences, Molecular Biotechnology Center, University of Turin, Via Nizza 52, I-10126 Turin, Italy; 5 Department of Molecular Cellular and Animal Biology, University of Camerino, Via Camerini 5, Camerino, Italy; 6 Division of Surgical Oncology, Department of Surgery, University of California, 10833 Le Conte Avenue, Los Angeles, CA 90095, USA; 7 CESI Aging Research Center, G D’Annunzio University, Via Colle dell’Ara, I-66013 Chieti Scalo, Chieti, Italy

## Abstract

**Introduction:**

The availability of mice transgenic for the human *HER2* gene (huHER2) and prone to the development of HER2-driven mammary carcinogenesis (referred to as *FVB-huHER2 mice*) prompted us to study active immunopreventive strategies targeting the human HER2 molecule in a tolerant host.

**Methods:**

FVB-huHER2 mice were vaccinated with either IL-12-adjuvanted human HER2-positive cancer cells or DNA vaccine carrying chimeric human-rat HER2 sequences. Onset and number of mammary tumors were recorded to evaluate vaccine potency. Mice sera were collected and passively transferred to xenograft-bearing mice to assess their antitumor efficacy.

**Results:**

Both cell and DNA vaccines significantly delayed tumor onset, leading to about 65% tumor-free mice at 70 weeks, whereas mock-vaccinated FVB-huHER2 controls developed mammary tumors at a median age of 45 weeks. In the DNA vaccinated group, 65% of mice were still tumor-free at about 90 weeks of age. The number of mammary tumors per mouse was also significantly reduced in vaccinated mice. Vaccines broke the immunological tolerance to the huHER2 transgene, inducing both humoral and cytokine responses. The DNA vaccine mainly induced a high and sustained level of anti-huHER2 antibodies, the cell vaccine also elicited interferon (IFN)-γ production. Sera of DNA-vaccinated mice transferred to xenograft-carrying mice significantly inhibited the growth of human HER2-positive cancer cells.

**Conclusions:**

Anti-huHER2 antibodies elicited in the tolerant host exert antitumor activity.

## Introduction

The ErbB-2 oncogene (HER2 in humans) is amplified and overexpressed in 20% to 30% of aggressive breast cancers, as well as in fractions of tumors of the ovary, stomach and others [1-3]. HER2 codes for a membrane tyrosine kinase; therefore, it can be targeted by antibodies and immune effector cells. Its oncogenic function, coupled to membrane expression, led to the definition of an oncoantigen [4,5], that is, an oncogene which is also a tumor-associated antigen that can be successfully targeted for tumor prevention and therapy. HER2-targeted therapies based on monoclonal antibodies (such as trastuzumab and pertuzumab) and on small-molecule inhibitors are in widespread clinical use. Even though generally well-tolerated, such treatments have some drawbacks: cost, need for continuous treatment, increased risk of cardiac toxicity and, mainly, frequent onset of resistance to therapy. The search for anti-HER2 vaccines able to break the patient’s tolerance toward HER2 is being pursued to elicit effective antitumor immune responses and immunological memory [6-8]. Anti-HER2 vaccines could be employed in a therapeutic setting, even in combination with other therapies, but most likely could be used in an adjuvant setting to prevent the development of metastases or to target the early stages of the disease, such as *in situ* carcinoma [9].

Preclinical studies on the prevention of mammary carcinogenesis driven by the rat HER2 gene (normal or mutated) have shown that several immune approaches can hamper the neoplastic process, ranging from the administration of cytokines, such as interleukin 12 (IL-12) [10], to active vaccination approaches [4,5]. Cell vaccines have shown high potency only when coupled with strong adjuvant stimuli (such as allogeneic stimulation and IL-12) [11,12]. Cytokines other than IL-12 were found to be far less efficient [12]. The adjuvant effect of IL-12 in cell vaccine also was found to be effective in curing minimal residual disease [13]. DNA vaccines also have proved to be highly effective [14-16].

Although passive targeting of the human HER2 (huHER2) homologue—for example, with antibodies—can easily be studied *in vivo* against human HER2-positive cancers grown as xenografts in immunodeficient mice, active immune approaches require immunocompetent mice tolerant to huHER2. Some huHER2 transgenic lines [17,18] were found not to develop spontaneous mammary tumors and were used only in vaccination challenge experiments to study the efficacy of anti-HER2 vaccines. Tumor-prone mice transgenic for huHER2, obtained by Finkle and co-workers [19], allow the study of immunoprevention of autochthonous tumor onset through vaccines targeting the normal huHER2 molecule. These mice (referred to as *FVB-huHER2* herein) carry a wild-type huHER2 gene under the control of the mouse mammary tumor virus (MMTV) promoter and show spontaneous development of mammary tumors in most female mice in the second semester of life. A few data have been reported on the prevention of tumors of these mice by passive transfer of antibodies [19] and by an anti-idiotype active immune approach [20].

In our present study, we used FVB-huHER2 transgenic mice to study anti-huHER2 vaccine strategies (a xenogeneic whole-cell vaccine and a DNA human/rat chimeric vaccine) to break tolerance to huHER2. We had two aims: (1) to evaluate vaccine efficacy in the immunoprevention of huHER2-driven mammary tumors, and (2) to study whether anti-HER2 antibodies resulting from a break of tolerance could inhibit human tumors growing as xenografts.

## Methods

### Mice

FVB-huHER2-transgenic mice were obtained from Genentech (line MMTV.f.hu.HER2#5(Fo5) on FVB background; South San Francisco, CA, USA) [19]. They carry the full-length, normal huHER2 gene under the control of the MMTV promoter. FVB-huHER2 mice were bred in our animal facilities and genetically screened by PCR using a primer set specific to human growth hormone exons 4 and 5, which are included in the transgene backbone, as reported previously [19]. Mice were inspected weekly by palpation. Progressively growing masses larger than 0.3 cm in diameter were scored as tumors. The mice were killed when the diameter of one of the tumors exceeded 1.7 cm. Nontransgenic FVB/NCrl (FVB) female mice were purchased from Charles River Laboratories (Calco, Como, Italy). For xenograft experiments, we used the immunodeficient Rag2^−/−^;Il2rg^−/−^ mice (kindly provided by Drs Nomura and Ito, Central Institute for Experimental Models, Kawasaki, Japan) [21]. *In vivo* experiments were performed in compliance with the Italian and European guidelines and were approved by the Institutional Review Board of the University of Bologna.

### Cells

The HER2-positive human ovarian carcinoma cell line SK-OV-3 was cultured in RPMI 1640 medium (Invitrogen, Milan, Italy) supplemented with 10% fetal bovine serum (FBS) and maintained at 37°C in a humidified atmosphere with 5% CO_2_. Other human cell lines with different HER2 expression were used as well: MDA-MB-453 (breast cancer origin, medium to high HER2 expression) [21], MCF-7 (breast cancer origin, low HER2 expression) and SJ-RH4 (rhabdomyosarcoma, null HER2 expression) [22]. We established a cell line, which we refer to as *syn-HER2*, from a mammary carcinoma of a FVB-huHER2 mouse. This cell line showed high expression of huHER2 and tumorigenicity in syngeneic hosts.

### Plasmids

Chimeric human/rat HER2 plasmid electroporated vaccine (HuRT), previously described in detail [16], derived from pVAX1 (Invitrogen), encodes a chimeric protein in which the first 390 extracellular NH_2_-terminal residues are from huHER2 and the remaining extracellular and transmembrane residues from rat HER2/neu. Empty vector pVAX1 was used as an experimental control. Large-scale production and purification of the plasmids were performed with EndoFree Plasmid Giga kits (QIAGEN, Valencia, CA, USA) as previously reported [15].

### Cytokine and vaccine treatments

The cell vaccine was formulated as mitomycin C–treated huHER2-overexpressing human cancer cells (SK-OV-3) associated with exogenous administration of recombinant murine IL-12 to provide the specific huHER2 antigen combined with adjuvants (xenogenicity and IL-12). The cell vaccination schedule was reported previously. Briefly it was based on a 4-week cycle. During the first 2 weeks, mice received four twice-weekly intraperitoneal (i.p.) vaccinations with 2 × 10^6^ proliferation-blocked (mitomycin C–treated [23]) SK-OV-3 cells in 0.4 ml of phosphate-buffered saline (PBS). During the third week, mice received daily i.p. administration of murine IL-12 followed by 1 week of rest [11]. The IL-12 dose was 50 ng per day in the first vaccination cycle and 100 ng per day in subsequent cycles. Vaccination cycles were repeated for the entire lifetime of the mouse. Control groups consisted of untreated mice and mice treated with vehicle alone (PBS). Mice were monitored weekly for mammary tumor onset.

DNA vaccination consisted of two intramuscular (i.m.) injections of 50 μg of plasmid diluted to a final volume of 40 μl per mouse in final concentrations of 0.9% NaCl and 6 mg/ml polyglutamate. Anesthetized mice received the injection of DNA vaccine into the tibial muscles (20 μl in each muscle) through a 28-gauge needle syringe. Immediately thereafter the muscle tissues were subjected to electroporation, consisting of two square wave, 25-ms, 375 V/cm pulses generated with a T830 electroporator (BTX, San Diego, CA, USA). The vaccination course consisted of two i.m. injections repeated at 14-day intervals according to the following schedule: first week, DNA vaccine; second week, rest; third week, DNA vaccine; and fourth to tenth weeks, rest [15]. Vaccinations were repeated for the entire lifetime of the mouse. Control groups consisted of untreated mice or mice treated with pVAX1 empty vector.

### Antibody response

Serum samples from vaccinated and control mice were collected periodically and stored frozen at −80°C. Anti-huHER2 antibodies were then detected by a specific enzyme-linked immunosorbent assay (ELISA) as described previously [15]. Thermo Scientific Immunoplate Nunc Maxisorp 96-well microplates (Cole-Parmer North America, Vernon Hills, CA, USA) were coated with the extracellular domain of huHER2 molecule, at 1 μg/ml and 100 μl/well, by overnight incubation. After blocking and washing incubations, sera at 1:250 to 1:500 dilutions were added. Secondary goat anti-mouse immunoglobulin G (IgG)-peroxidase conjugate antibody (1:12,000 dilution; Calbiochem, San Diego, CA, USA) was added after plate washing. Next, 100 μl of 3,3′,5,5′-tetramethylbenzidine peroxidase substrate were added (Thermo Scientific, Rockford, IL, USA). After the reaction was stopped with 0.18 M sulfuric acid, absorbance was measured at 450 nm and 620 nm using an ELISA microreader (Tecan Systems, San Jose, CA, USA). A standard curve with anti-huHER2 murine monoclonal antibody clone 4D5 (Genentech) was run in parallel (0.04 to 30 ng/ml). Anti-HER-2/*neu* total antibodies and subclasses were studied by flow cytometry as reported previously [11].

### Cytokine production

Spleen cells were collected from vaccinated and control mice after at least three vaccination cycles. Interferon γ (IFN-γ) production by spleen mononuclear cells was evaluated after *in vitro* culture for 6 days alone (spontaneous release) or in the presence of proliferation-blocked huHER2-positive cells (at a 10:1 lymphocyte/tumor cell ratio) in RPMI 1640 medium supplemented with 10% FBS and recombinant IL-2 (20 U/ml) as described previously [11]. HuHER2-positive cells used were a cell line derived from mammary cancer of FVB-huHER2 (referred to as *syn-HER2*) and human SK-OV-3 cells (referred to as *xeno-HER2*). Culture supernatants were collected, and murine IFN-γ was quantified by ELISA (R&D Systems, Minneapolis, MN, USA).

### Whole-mount tissue sections

Whole-mount sections of all mammary glands were prepared as described previously [24]. Briefly, mouse skin was removed and fixed overnight in 10% buffered formalin. Mammary fat pads were scored into quarters, gently scraped from the skin and immersed in acetone overnight. After rehydration, samples were stained with ferric hematoxylin (Sigma-Aldrich, Milan, Italy), dehydrated in increasing concentrations of alcohol, cleared with limonene and stored in methyl salicylate (Sigma-Aldrich). Digital pictures were taken with a Nikon COOLPIX 995 camera (Nikon Europe, Turin, Italy) mounted on a stereoscopic microscope (Leica MZ6; Leica Microsystems, Buffalo Grove, IL, USA).

### Immunohistochemistry

Optimal cutting temperature compound–embedded sections were immunostained with the following antibodies: CD4 (rat anti-mouse; BD Biosciences Pharmingen, San Diego, CA, USA), CD8 (rat anti-mouse; BD Biosciences Pharmingen), CD11b (rat anti-mouse; BD Biosciences Pharmingen), CD68 (rat monoclonal antibody; Abcam, Cambridge, UK), FoxP3 (rat anti-mouse; eBioscience, San Diego, CA, USA), Gr-1 (rat anti-mouse; BD Biosciences Pharmingen), B220 (rat anti-mouse; BD Biosciences Pharmingen) and CD31/105 (rat anti-mouse; BD Biosciences Pharmingen) for vessels. After being washed, the sections were overlaid with appropriate secondary antibodies. Immunostaining was developed using the chromogen 3-amino-9-ethylcarbazole in the LabVision Ready-to-Use AEC Substrate System (Thermo Scientific) or a standard streptavidin biotinylated alkaline phosphatase method (Thermo Scientific).

### HER2 signaling

Cells were lysed with Novagen PhosphoSafe Extraction Reagent (EMD Millipore, Milan, Italy) plus phosphatase and protease inhibitors (purchased from Sigma-Aldrich) and incubated for 10 minutes at room temperature. Nuclei were removed by centrifugation at 12,000 × *g* at 4°C for 15 minutes, and the protein concentration in the supernatants was determined by DC Protein Assay (Bio-Rad Laboratories, Milan, Italy) using bovine serum albumin as the standard. Proteins were separated on an 8% polyacrylamide gel (20 μg of total lysate), then transferred to polyvinylidene difluoride membranes (Bio-Rad Laboratories). After blocking with PBS containing 0.1% Tween 20 plus 5% nonfat dry milk for 2 hours at room temperature, membranes were incubated overnight at 4°C with primary antibodies diluted in blocking buffer. Anti-c-ErbB2/c-Neu (Ab3) mouse monoclonal antibody (3B5) (0.2 μg/ml; Calbiochem/EMD Chemicals, San Diego, CA, USA), anti-p-Neu (Tyr 1248)-R rabbit polyclonal antibody (0.2 μg/ml sc-12352-R; (Santa Cruz Biotechnology, Santa Cruz, CA, USA), anti-AKT rabbit polyclonal antibody (1:1,000 dilution; 9272), anti-phospho-Akt (Ser473) (D9E) XP rabbit monoclonal (1:1,000 dilution; 4060) (all purchased from Cell Signaling Technology, Danvers, MA, USA) and anti-actin rabbit antibody (1 μg/ml; Sigma-Aldrich) were used as primary antibodies. After incubation with the respective horseradish peroxidase–labeled secondary antibodies (Santa Cruz Biotechnology), protein presence was revealed by chemiluminescence reaction (LiteAblotplus chemiluminescence substrate; EuroClone, Milan, Italy).

### Adoptive transfer of spleen cells from vaccinated mice

Mice received two vaccinations with HuRT-DNA or pVAX1 (as reported above) at 2-week intervals, then spleen cells were collected and coinjected subcutaneously (s.c.) with Syn-HER2 cancer cells. Tumor diameter was measured twice weekly with digital calipers. Tumor volumes were calculated as π/6•[√(*a*•*b*)]^3^, where *a* = maximal tumor diameter and *b* = major tumor diameter perpendicular to *a*.

### HER2-positive xenograft therapy

The therapeutic activity of anti-huHER2 antibodies elicited by HuRT-DNA in FVB-huHER2 mice was tested against HER2-positive human xenografts. Groups of five to seven Rag2^−/−^;Il2rg^−/−^ female mice received i.p. injections of 2 × 10^6^ SK-OV-3 ovarian carcinoma cells. Starting on the following day, mice received i.p. 200 μl of pooled sera from mice vaccinated with HuRT-DNA. Treatment was repeated on days 3, 7 and 14 afterward. Control mice received sera pooled from control FVB-huHER2 mice (untreated or pVAX1-treated). The mice were killed 5 weeks after cancer cell injection. Accurate necropsy was performed, and intraperitoneal tumor masses were collected and weighed to quantify therapeutic efficacy.

### Statistical analysis

The logrank Mantel–Haenszel test was used to compare tumor-free survival curves. Student’s *t*-test and a Wilcoxon nonparametric test were used for other comparisons.

## Results

### HER2-transgenic model and vaccines

The mouse model of mammary carcinogenesis driven by the wild-type huHER2 oncogene (referred to as *FVB-huHER2* herein) [19] is characterized by tumor onset after more than 40 weeks of age and by low numbers of neoplastic mammary glands, similar to what happens in mice transgenic for the wild-type rat HER2 homologue [10,11,25].

From previous experiments on immunoprevention of rat neu-driven carcinogenesis [5], we learned that both a whole-cell vaccine and a DNA vaccine can be effective, and that efficacy depends on vaccine conditions and the level of antibody response [12,23]. Therefore, we performed preliminary experiments to choose vaccines and conditions able to induce the highest anti-huHER2 antibody response.

**Figure 1:**
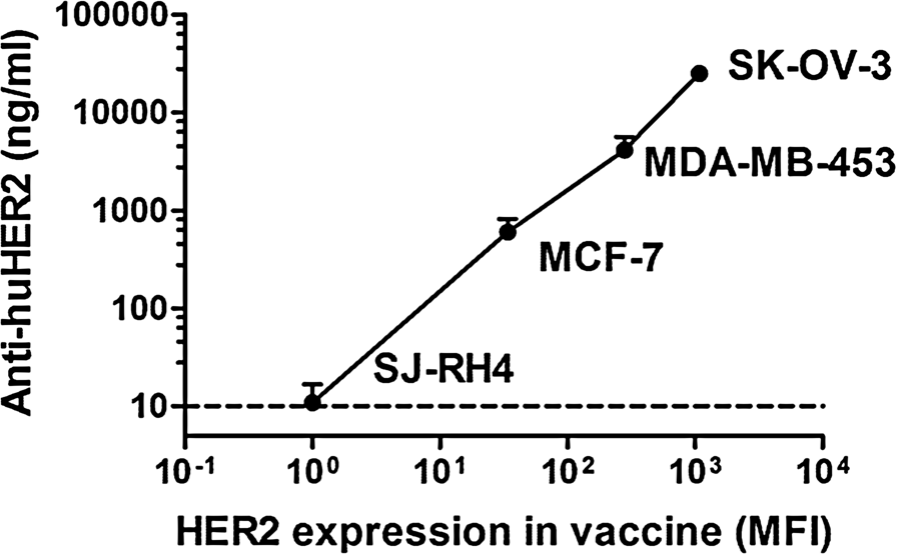
**Induction of anti-huHER2 antibody response by human cancer cell vaccines with different levels of HER2 membrane expression.** Nontransgenic mice received one cycle of vaccination (cells and recombinant interleukin 12) as reported in Methods. The *x*-axis represents the HER2 expression level (cytofluorometric evaluation by mean fluorescence intensity (MFI) measured in arbitrary units) of cells used for vaccinations, and the *y*-axis represents the corresponding level of anti-huHER2 antibodies elicited by vaccinations. The dashed line shows the enzyme-linked immunosorbent assay detection sensitivity limit. Data shown are the mean and SEM from five mice per group. huHER2, human *HER2* gene.

To choose the best whole-cell vaccine, we vaccinated nontransgenic mice with a panel of human tumor cell lines with different HER2 expression, using IL-12 as a biological adjuvant. The level of anti-huHER2 antibodies elicited was proportional to the log-transformed membrane expression of HER2 (Figure 1). To maximize antibody induction in tolerant FVB-huHER2-transgenic mice, we therefore chose the SK-OV-3 cell line as the cell vaccine (that is, the highest inducer of anti-huHER2 antibodies together with recombinant IL-12 (hereafter referred to as the *HER2*-*cell vaccine*). DNA cell vaccine was a chimeric human/rat HER2 construct (HuRT-DNA vaccine), which was previously reported to be a good inducer of anti-HER2 antibodies [16,18].

Regarding the vaccination schedule, we previously found in rat HER2/neu-transgenic mice that a fast, high induction of anti-HER2/neu antibodies was required to obtain the highest cancer immunopreventive activity [23,26]. To set up the optimal time to start vaccination of transgenic FVB-huHER2 mice, we vaccinated mice of different ages and examined the anti-huHER2 antibody levels obtained 4 weeks after the first vaccination (Table 1). We found that the response to the HuRT-DNA vaccine was dependent on the age at first vaccination, with the maximum level reached at 18 to 20 weeks of age. The humoral response to HER2-cell vaccine reached levels lower than DNA vaccine and was not related to age. Therefore, we decided to start HER2-cell vaccine treatment as soon as possible (6 weeks of age) and HuRT-DNA vaccine at the optimal time for anti-huHER2 antibody induction (18 weeks of age).

**Table 1:** **Vaccination of FVB-huHER2 mice started at different ages: effect on induction of anti-huHER2 antibody levels at 4 weeks after first vaccination**^**a**^

**Vaccine**	**Start of vaccination (weeks of age)**	**Number of mice**	**Anti-huHER2 antibodies (ng/ml), mean ± standard error**
HER2-cell	5 to 7	9	1,121 ± 347
	8 to 12	6	720 ± 327
	18 to 20	4	692 ± 243
	24 to 26	6	1,107 ± 123
HuRT-DNA	11 to 15	6	1,671 ± 440*
	18 to 20	6	5,297 ± 1,284
	24 to 26	5	667 ± 277*

^a^FVB-huHER2, FVB mice transgenic for huHER2; huHER2, human *HER2* gene. *P = 0.02 vs. HuRT-DNA at 18 to 20 weeks (Student’s *t*-test).

### Immunoprevention of huHER2-driven mammary carcinoma

To prevent the onset of mammary carcinoma, FVB-huHER2 mice received lifelong vaccinations with HER2-cell or HuRT-DNA anti-human HER2 vaccines. The experimental end points were the age at the onset of the first mammary carcinoma and the total number of tumors per mouse.

**Figure 2:**
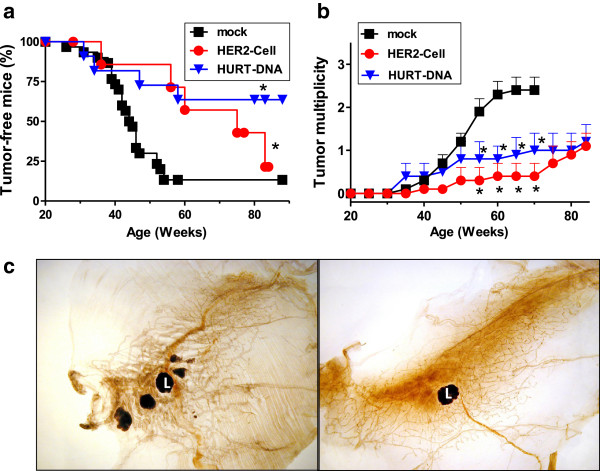
**Inhibition of mammary carcinogenesis in FVB-huHER2 mice by anti-HER2 vaccines. (a)** Tumor-free survival (*P < 0.05 vs. mock group by Mantel-Haenszel analysis). **(b)** Tumor multiplicity (mean number ± SEM) (*P < 0.05 vs. mock vaccination group by Student’s *t*-test). **(c)** Whole-mount section of the fourth mammary gland of control (left panel) or vaccinated (right panel) mice at 60 weeks of age. L = lymph node. Mock: The results in the control groups (untreated, vehicle and pVAX1) were not significantly different, and the data were cumulated (*n* = 31). HER2-cell vaccine (*n* = 8); HuRT-DNA vaccine (*n* = 12). FVB-huHER2, FVB mice transgenic for huHER2; HuRT, chimeric human/rat HER2 plasmid electroporated vaccine.

Both vaccines significantly delayed tumor onset in FVB-huHER2 mice, with about 65% of mice being tumor-free at 70 weeks of age (*P* < 0.05 by logrank Mantel–Haenszel test), whereas the median latency time of tumors was 45 weeks in mock-vaccinated mice (Figure 2a). In HuRT-DNA-vaccinated mice, even at about 90 weeks, 65% of mice were tumor-free. Vaccines significantly decreased tumor multiplicity compared to controls (*P* < 0.05 by Student’s *t*-test) (Figure 2b). The median times from tumor onset to death were 11.5 weeks for mock-vaccinated mice, 16 weeks for HER2-cell-vaccinated mice and 14.5 weeks for HuRT-DNA-vaccinated mice. The data show that vaccinations almost doubled the lifespans of the mice.

Whole-mount tissue section analysis of mammary glands showed multifocal carcinogenesis in control mice (Figure 2c, left) with focal huHER2 expression in ducts fostering the occurrence of neoplastic lesions, whereas mammary glands of HuRT-DNA-vaccinated mice were almost devoid of neoplastic lesions (Figure 2c, right).

### Immune responses elicited by vaccines

**Figure 3:**
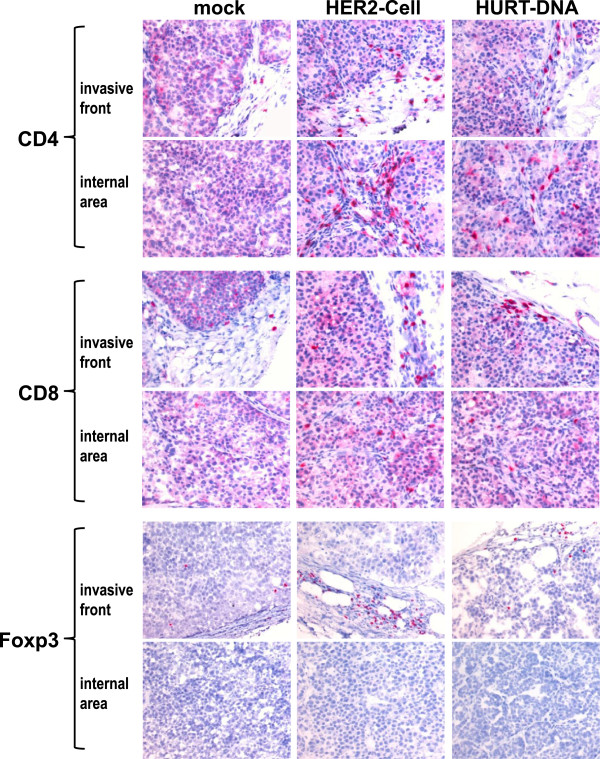
**Immunohistochemical staining of tumors grown in mock-, HER2-cell- and HuRT-DNA-vaccinated mice.** Red staining shows positivity for the indicated molecule. Original magnification × 400. HuRT, chimeric human/rat HER2 plasmid electroporated vaccine.

**Figure 4:**
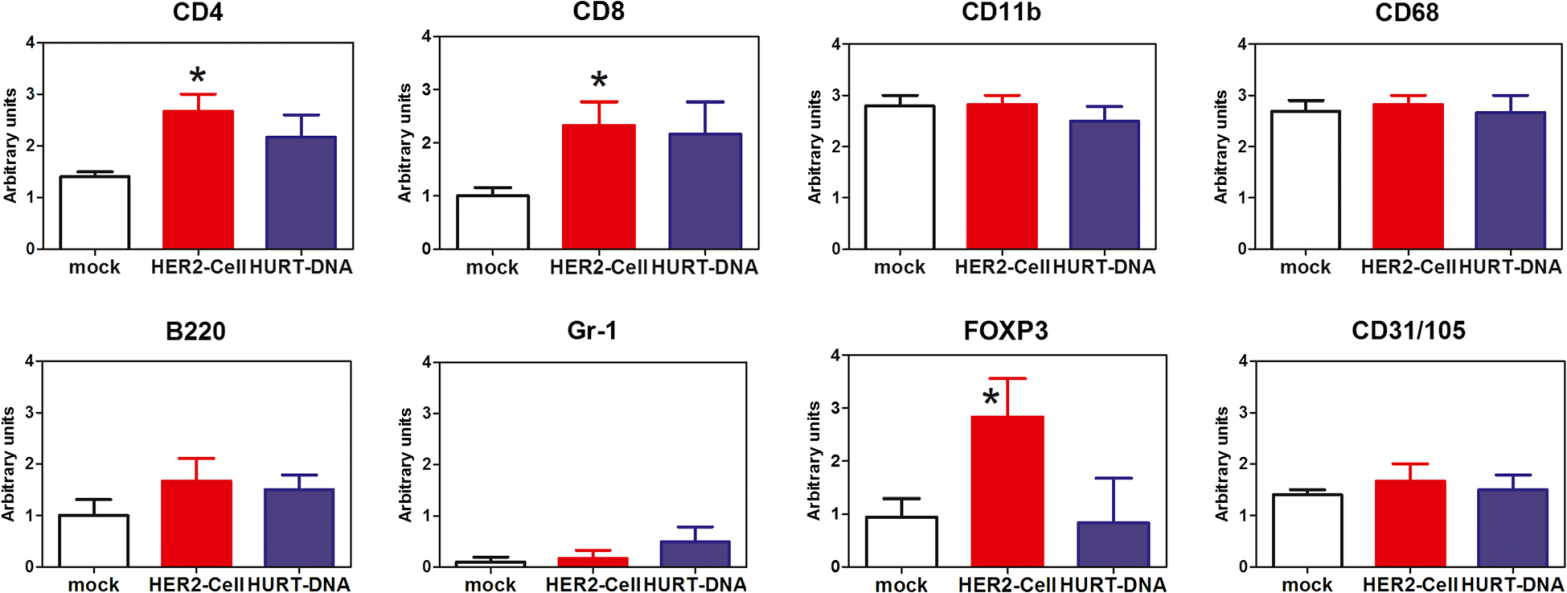
**Semiquantitative analysis of the populations infiltrating tumors grown in mock-, HER2-cell- and HuRT-DNA-vaccinated mice.** Mean and SEM of three to nine mice per group. *P < 0.05 vs. mock-vaccinated control group by Student’s *t*-test. HuRT, chimeric human/rat HER2 plasmid electroporated vaccine.

**Figure 5:**
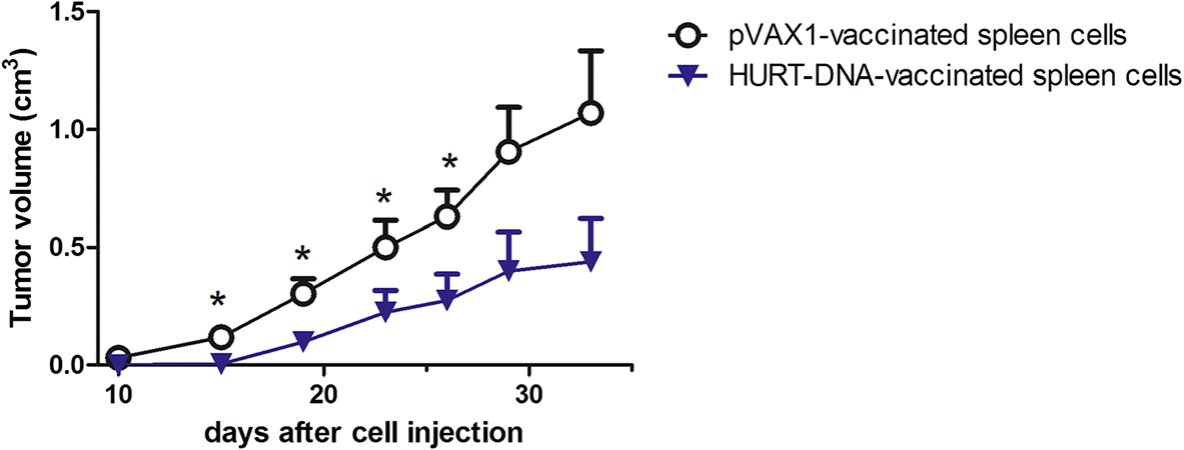
**Activity of adoptively transferred spleen cells from mice vaccinated with empty vector (pVAX1) or with HuRT-DNA against the subcutaneous growth of syn-HER2 cancer cells (Winn test).** A 40:1 ratio of spleen to cancer cells was used. Data are mean ± SEM of five mice per group. *P < 0.05 vs. pVAX1-vaccinated group by Student’s *t*-test. HuRT, chimeric human/rat HER2 plasmid electroporated vaccine.

The immunohistochemical staining of tumors grown in control and vaccinated mice showed a marked increase of lymphocyte infiltration in the two vaccinated groups. Figure 3 shows that though only a few CD4+ and CD8+ lymphocytes and rare Foxp3 cells were present at the edges of control group tumors, numerous CD4+ and CD8+ cells were found in both the external and inner parts of the tumors in vaccinated groups. Interestingly, the HER2-cell-vaccinated group also showed a significant increase in Foxp3-positive regulatory T cells (Figure 4). No significant differences were noted in the myeloid infiltrate (CD11b+, CD68+ and Gr-1+) or in B cells (Figure 4). To functionally assess the overall activity of leukocyte responses elicited by anti-HER2 vaccines, we coinjected syn-HER2 tumor cells and spleen cells into syngeneic mice. In this adoptive transfer experiment, the splenocytes of HuRT-DNA vaccinated mice delayed tumor growth in comparison to splenocytes of mock-vaccinated (pVAX1) mice (Figure 5).

**Figure 6:**
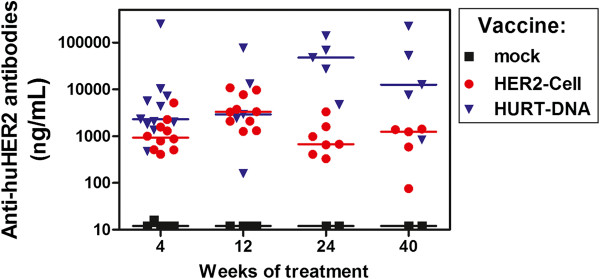
**Serum anti-huHER2 antibodies induced by periodic vaccination of FVB-huHER2 mice with HER2-cell or HuRT-DNA.** See Methods section for details on vaccination cycles. Horizontal bar represents the median value of each group. HuRT-DNA vaccine group had significantly higher antibody levels than the HER2-cell vaccine group at 4, 24 and 40 weeks of treatment (*P* < 0.05 by Wilcoxon nonparametric test). HuRT, chimeric human/rat HER2 plasmid electroporated vaccine.

Both vaccines broke the tolerance toward huHER2 and rapidly elicited anti-huHER2 humoral responses, with some differences in potency and kinetics between vaccines, which are in agreement with preliminary experiments. The HuRT-DNA vaccine elicited a very high, steady level of antibodies, reaching 20 to 50 μg/ml, as shown by a specific ELISA (Figure 6), whereas antibodies induced by the HER2-cell vaccine peaked at 12 weeks and decreased thereafter with a plateau at about 1 μg/ml.

**Figure 7:**
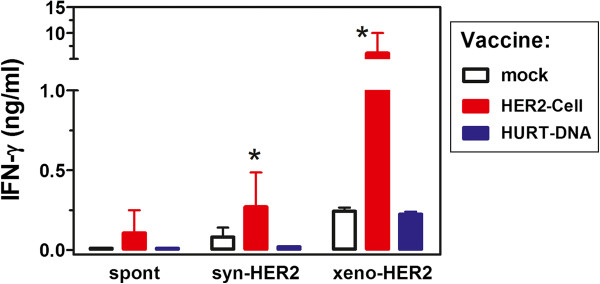
**Interferon γ production by spleen cells of FVB-huHER2-vaccinated mice.** Mean ± SEM data are shown for each group (*n* = 3 to 6 mice per group). Spleen cells were cultured alone (spont) or in the presence of huHER2-positive cancer cells of syngeneic (syn-HER2) or xenogeneic (xeno-HER2) origin. *P ≤ 0.05 vs. other groups. HuRT, chimeric human/rat HER2 plasmid electroporated vaccine; IFN, interferon.

IFN-γ production by spleen cells was studied in vaccinated and control mice (Figure 7). Splenocytes of HER2-cell-vaccine-treated mice spontaneously released IFN-γ, the amount of which was significantly increased after *in vitro* culture with syngeneic HER2-positive cells (syn-HER2) and reached very high levels after culture with vaccine cells (xeno-HER2). Such high *in vitro* response to xeno-HER2 cells could be also attributed to a xenogeneic reaction to human antigens unrelated to HER2, thus mimicking the IFN-γ burst that happened *in vivo* after each vaccination cycle. Splenocytes from HuRT-DNA vaccinated mice did not release IFN-γ, and a low IFN-γ response to huHER2-positive cells was observed. The DNA vaccine was as effective as (or even more effective than) the HER2-cell vaccine for immunoprevention, even in the absence of an IFN-γ burst, thus suggesting that a high level of anti-HER2 antibodies could be sufficient to prevent tumor onset.

**Figure 8:**
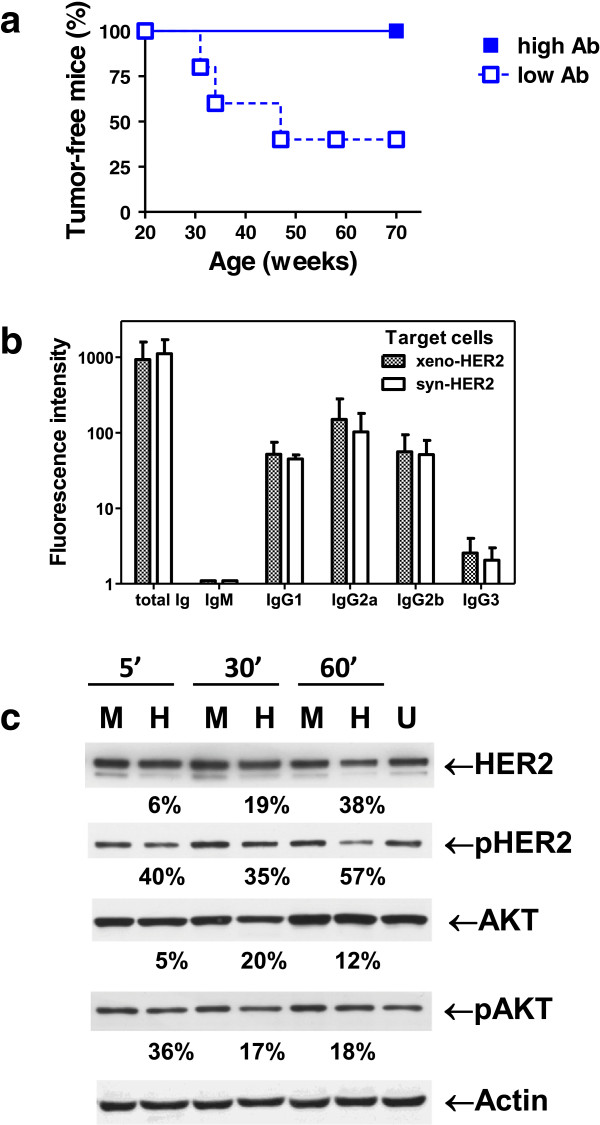
**Anti-huHER2 antibodies elicited by HuRT-DNA. (a)** Tumor-free survival in mice vaccinated with chimeric human/rat HER2 plasmid electroporated vaccine (HuRT)-DNA stratified according to anti-huHER2 levels above (high antibody (Ab) level) or equal to or below (low antibody level) the median after the first vaccination cycle (*n* = 5 mice per group). *P* < 0.05 between the two groups by Mantel-Haenszel test. **(b)** Isotypes of anti-HER2 antibodies. Sera were incubated with syngeneic or xenogeneic target cells (syn-HER2 and xeno-HER2, respectively), then indirect immunofluorescence was performed with fluorescein-labeled secondary antibodies to detect murine immunoglobulin (total Ig) or specific murine isotypes. **(c)** Anti-huHER2 antibodies elicited by HuRT-DNA caused a decreased huHER2 signaling in syn-HER2 cells. U = untreated cells; M = cells treated for the indicated times with sera from Mock-vaccinated mice; H = cells treated for the indicated times with sera from mice vaccinated with HuRT-DNA. Time of exposure to antisera is reported at the top of the figure. Densitometric analysis was performed. Band intensity was normalized over the corresponding actin, then the percentage decrease between M and H after the same incubation time was calculated and is shown under the corresponding H lane.

To further investigate the antitumor activity of anti-huHER2 antibodies, we focused on the HuRT-DNA vaccine because it induced higher titers of anti-huHER2 antibodies and was devoid of extraneous xenogeneic stimuli that could elicit responses against antigens other than HER2. The level of anti-huHER2 antibodies after the first vaccination cycle was used to stratify mice into two groups: above and equal to or below median levels. A significant difference in tumor-free survival was observed between the two groups (Figure 8a), thus confirming that, in huHER2-transgenic mice, cancer prevention required a fast, high antibody response. Study of the antibody isotypes showed that IgG1, IgG2a and IgG2b were well-represented in the sera of vaccinated mice and that both type 1 IgG2 and type 2 IgG1 recognized syn-HER2 and xeno-HER2 cancer cells with identical isotype patterns (Figure 8b). Anti-huHER2 antibodies affected signaling events downstream of HER2. Treatment of syn-HER2 cancer cells with HuRT-DNA-induced antisera caused a significant decrease in pHER2 and pAKT (Figure 8c).

### Antibodies elicited in tolerant hosts impair the growth of human HER2-positive tumor cells *in vivo*

The results demonstrate that anti-human HER2 vaccines elicited protective immune responses against mouse tumors expressing huHER2. To further extend the scope of these studies, we sought to determine whether the antibodies elicited by our vaccines could also inhibit the *in vivo* growth of human cancers expressing HER2.

**Figure 9:**
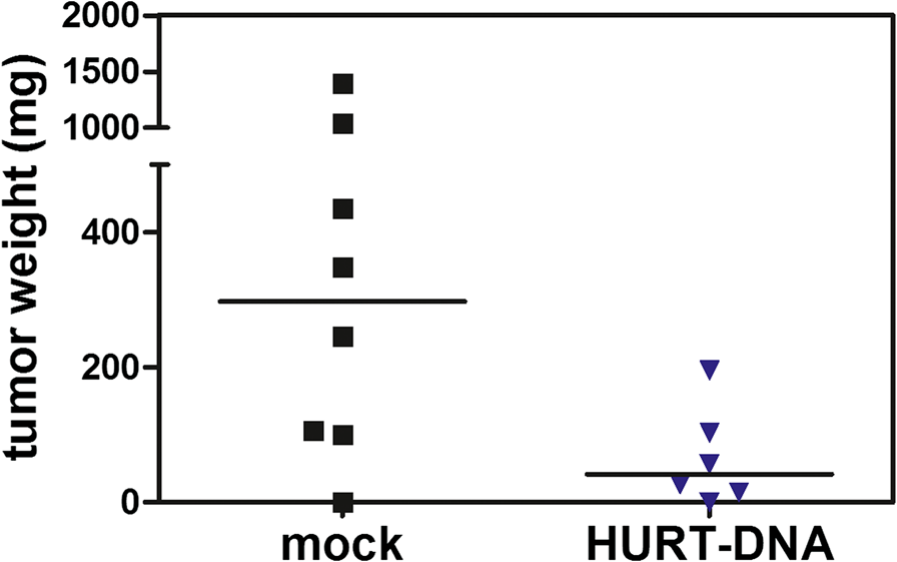
**Inhibition of HER2-positive SK-OV-3 xenograft intraperitoneal growth in immunodeficient mice injected intraperitoneally with sera from FVB-huHER2-vaccinated mice.** Each point represents the weight of all tumor masses grown intraperitoneally and dissected from each mouse. Horizontal bar represents the median value of each group. *P* = 0.05 by Wilcoxon nonparametric test. HuRT, chimeric human/rat HER2 plasmid electroporated vaccine.

The antitumoral effectiveness of high-level anti-huHER2 antibodies elicited by HuRT-DNA vaccine in tolerant hosts was therefore tested according to their ability to hamper the growth of human HER2-positive cancer cells in immunodeficient mice. Rag2^−/−^;Il2rg^−/−^ mice receiving i.p. injections of human HER2-positive SK-OV-3 cancer cells developed peritoneal carcinomatosis that can be accurately measured by the collection and weighing of dissected tumor masses [21,27]. The weekly i.p. injection of sera from HuRT-DNA-vaccinated mice significantly decreased peritoneal carcinomatosis (Figure 9); therefore, anti-huHER2 antibodies elicited by DNA vaccine in tolerant hosts impair human HER2-positive cancer growth *in vivo* in immunodeficient mice, even in the absence of most cellular and cytokine responses.

## Discussion

The FVB-huHER2 mouse, which is prone to the development of autochthonous mammary tumors driven by huHER2 expression, was used as a model with which to study active immunological strategies targeting the human HER2 molecule in a tolerant host. Both a whole-cell vaccine and a DNA vaccine succeeded in breaking tolerance toward huHER2, eliciting high levels of anti-huHER2 antibodies and resulting in a significant delay in tumor onset. In addition to the prevention of huHER2-driven carcinogenesis in the original host, antibodies elicited by the HuRT-DNA vaccine inhibited the growth of human tumors when passively administered to immunodeficient mice bearing HER2-positive xenografts.

The first conclusion we can draw from these results is that vaccines against the human wild-type HER2 can break tolerance and efficiently inhibit mammary carcinogenesis driven by wild-type huHER2. Most cancer immunoprevention studies reported to date were conducted with mice transgenic for the rat homologue [5].

In the present work, the cell vaccine consisted of human HER2-positive cancer cells combined with IL-12 administration. Therefore, xenogeneic antigens and IL-12 had an adjuvant effect able to break tolerance to HER2, similarly to what we previously reported with regard to the combination of allogeneic antigens and IL-12 [11,12]. The HER2-cell vaccine elicited both anti-huHER2 antibodies and a strong IFN-γ response.

The DNA vaccine was engineered to encode a chimeric human-rat HER2 protein and was coupled to *in vivo* electroporation. Therefore, adjuvant effects of both xenogeneic HER2 sequences and electroporation broke the immunological tolerance to huHER2, resulting in a very high level of specific anti-huHER2 antibodies (in agreement with previous studies [16,18]) coupled with a very limited induction of IFN-γ. These results suggest that an anti-HER2 DNA vaccine and electroporation could be very advantageous in a clinical setting, yielding higher antibody responses than whole-cell vaccines (possibly as a result of lower local recruitment of Foxp3 regulatory T cells) without the risk of high IFN-γ stimulation, as also reported for work in different model systems [15]. In fact, data on the safety of DNA anti-HER2 vaccines have been reported in a pilot clinical trial [28].

The antitumor activity of anti-huHER2 antibodies elicited in a nontolerant host is well-known; in fact, trastuzumab and other monoclonal antibodies used in human therapy were originally obtained through immunization of mice against the xenogeneic human HER2 molecule. However, little is known about the antitumor activity of antibodies against huHER2 elicited in a tolerant host. Studies in rat HER2-transgenic mice have shown that the break of immunological tolerance to HER2 was based on the recognition of subdominant epitopes, because dominant clones had been selectively deleted [29]. A similar condition is likely to occur in humans, who are naturally tolerant to endogenous HER2.

Are antibodies induced by the break of tolerance able to inhibit tumor growth? We found that passive transfer of antisera from DNA-vaccinated, FVB-huHER2-transgenic mice to immunodeficient mice carrying xenografts of HER2-positive human cancer cells inhibited human tumor growth. The Rag2^−/−^;Il2rg^−/−^ immunodeficient mice used in our present study lacked the effectors mostly active in antibody-dependent cellular cytotoxic (ADCC) responses, (that is, natural killer (NK) cells), and could underestimate the potential antibody efficacy obtainable in immunocompetent hosts. In these mice, however, other cytotoxic antibody functions can be active, such as ADCC responses by leukocytes other than NK cells, complement-dependent cytotoxicity and direct effects on signaling downstream HER2.

## Conclusions

The FVB-huHER2-transgenic mouse is a unique model system that allows immunological studies of anti-human HER2 responses in immunocompetent, HER2-tolerant hosts prone to mammary carcinoma onset. The results reported herein show that effective vaccinations break the immunological tolerance to human HER2 and elicit immune responses, in particular antibodies, that inhibit the onset of murine HER2-positive mammary carcinoma and inhibit the *in vivo* growth of human HER2-positive cancer cells.

## Abbreviations

ADCC: antibody-dependent, cell-mediated cytotoxicity; huHER2: human HER2; HER2-cell: SK-OV-3 cell vaccine combined with recombinant interleukin 12; HuRT-DNA: chimeric human/rat HER2 plasmid electroporated vaccine; IL-12: interleukin 12; IFN-γ: interferon γ; PBS: phosphate-buffered saline; SEM: standard error of the mean.

## Competing interests

The authors declare that they have no competing interests.

## Authors’ contributions

CDG, PN and PLL designed the experiments, analyzed data and drafted the manuscript. AA, FC and EQ designed and produced the DNA vaccine. LL, AP, SC, MLI and MDO prepared the cell vaccines, studied the immune mechanisms and signaling and analyzed the results. MLP conceived and set up the original ELISA used to measure anti-HER2 antibodies and critically revised the manuscript. GN, VG, DR and RL performed *in vivo* studies, analyzed the results and revised the manuscript. MI performed histological and immunohistochemical analyses, interpreted the results and contributed relevant sections to the manuscript. All authors read and approved the final manuscript.
